# Genetics and therapy for pediatric eye diseases

**DOI:** 10.1016/j.ebiom.2021.103360

**Published:** 2021-05-08

**Authors:** Holly.Y. Chen, Ordan J. Lehmann, Anand Swaroop

**Affiliations:** a Neurobiology-Neurodegeneration & Repair Laboratory, National Eye Institute, National Institutes of Health, MSC0610, 6 Center Drive, Bethesda, MD 20892 USA; b Department of Ophthalmology and Visual Sciences, University of Alberta, Edmonton, Canada; c Department of Medical Genetics, University of Alberta, Edmonton, Canada

**Keywords:** Eye organogenesis, Congenital disorders, Ocular diseases, Vision impairment, Retinal development, Therapy

## Abstract

Ocular morphogenesis in vertebrates is a highly organized process, orchestrated largely by intrinsic genetic programs that exhibit stringent spatiotemporal control. Alternations in these genetic instructions can lead to hereditary or nonhereditary congenital disorders, a major cause of childhood visual impairment, and contribute to common late-onset blinding diseases. Currently, limited treatment options exist for clinical phenotypes involving eye development. This review summarizes recent advances in our understanding of early-onset ocular disorders and highlights genetic complexities in development and diseases, specifically focusing on coloboma, congenital glaucoma and Leber congenital amaurosis. We also discuss innovative paradigms for potential therapeutic modalities.

## 1 Introduction

Amongst the plethora of ocular disorders, congenital anomalies and early-onset diseases have particular significance. From a clinical perspective, these pediatric conditions frequently destine children to a lifetime of severe vision impairment and/or blindness, with long-lasting societal effects not least in terms of future employment. Equally, scientific importance of these largely heritable phenotypes is reflected by molecular entry points for characterizing developmental mechanisms relevant to the eye and frequently multiple other organ systems, as well as their substantial contribution to adult-onset diseases. Significance is further enhanced by the eye being an optically clear and uniquely accessible part of the central nervous system. These attributes facilitate phenotyping at the cellular level *in vivo* and have accelerated advancement of therapeutic approaches in multiple areas of medicine.

**Fig. 1:**
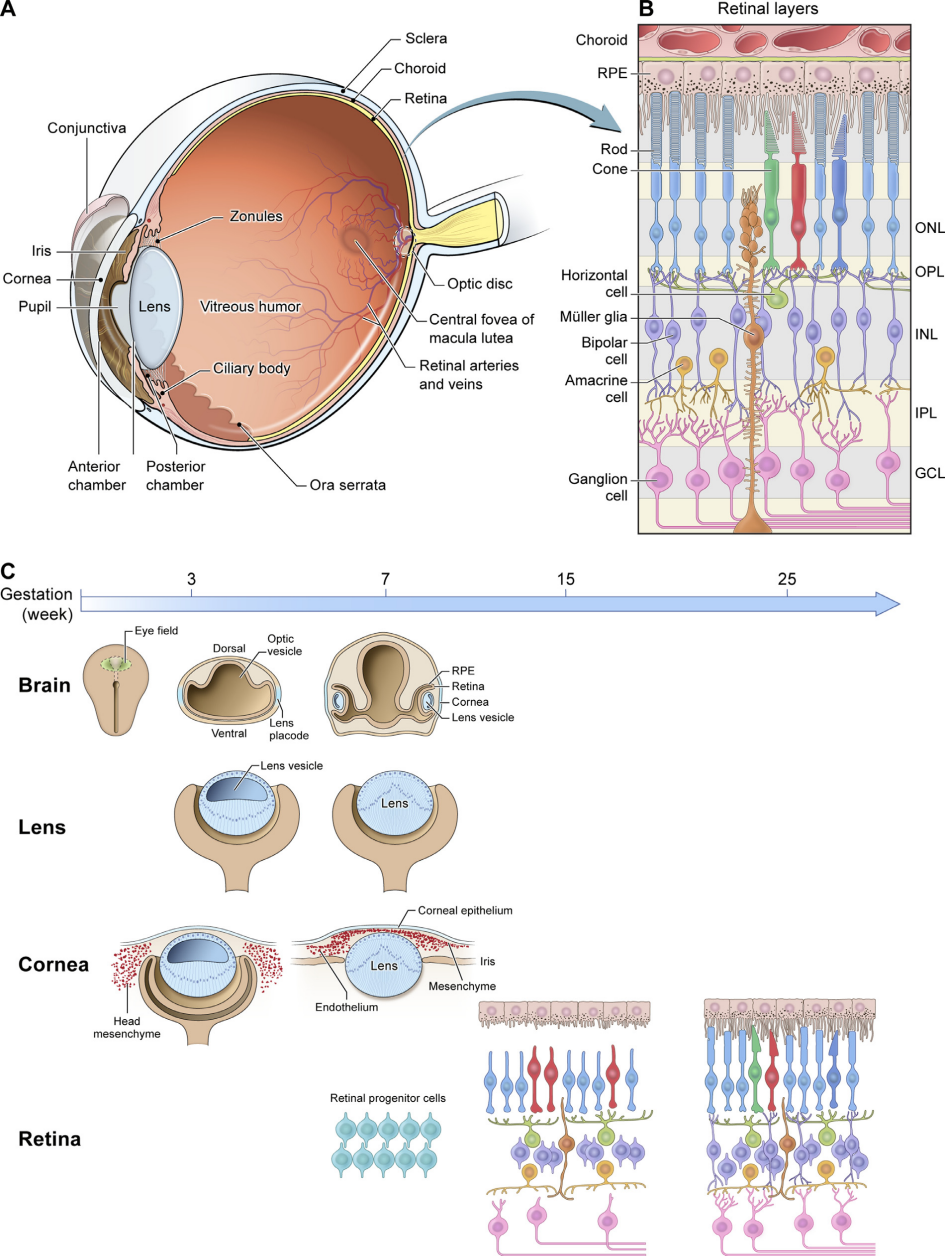
Cross-sectional images of the human eye (A) and the light-sensitive retina (B). (C) Development of the human eye. Eye field specification starts at around three weeks gestation in the anterior neural plate, followed by the formation of optic vesicle, lens, cornea and retina at various stages. ONL, outer nuclear layer; OPL, outer plexiform layer; INL, inner nuclear layer; IPL, inner plexiform layer; GCL, ganglion cell layer.

Appreciation of the mechanistic basis of pediatric disorders, presented here, is augmented by an understanding of key steps in eye development. In vertebrates, the eye's multiple components include the light-focusing cornea and lens of the anterior segment, and the posterior light-sensitive neural retina which captures, integrates and processes visual information before the optic nerve delivers it to the brain (Fig. 1A and 1B). First steps in human eye specification begin during the third and fourth week of gestation. These stages are stringently regulated, frequently in a reiterative manner, by molecular networks that include eye-field transcription factors as well as Hedgehog, Retinoic acid (RA), Wingless and int-1 (Wnt), Transforming growth factor beta (TGF-β), and other signaling pathways [1,2]. After initial specification of the eye field and its subsequent Hedgehog-mediated separation into two domains, the earliest morphological evidence of ocular development is the evagination of the optic vesicles from the prosencephalon (Fig. 1C) [1,3]. Patterning along a proximal-distal axis yields the optic stalk that connects the optic vesicle to the forebrain and eventually forms the optic nerve. The distal portion of the optic vesicle later invaginates to form a bilayered optic cup, and the neural retina and retinal pigment epithelium ultimately differentiate from these laminae.

Among other essential phases of eye development is the contact of the optic vesicle with the overlying surface ectoderm, triggering a sequence of inductive events: first generating the lens placode, then the lens vesicle, and ultimately the crystalline lens that focuses incident light onto the retina. Extensive remodeling is also required for the optic cup to form an intact spherical globe, a process that involves closure of a large fissure on the ventral or inferior portion of the eye. Failure of this choroid fissure to close results in coloboma (plural colobomata) – a spectrum of pediatric defects and malformations that represent important causes of visual impairment and blindness. The tightly regulated interval during which the fissure is open provides transient intraocular access for migrating neural crest cells [4]. These stem cells delaminate from edges of the cranial neural tube, and after a lengthy migratory route, envelop the eye and contribute to numerous extra- and intra-ocular structures. Consequently, perturbation of neural crest function results in optic fissure closure defects [5,6] as well as diverse congenital ocular diseases, including Axenfeld-Rieger syndrome and congenital glaucoma.

Another key facet of ocular development concerns the processes by which retinal progenitor cells (RPCs), derived from neuroepithelium of the optic cup, form six distinct neuronal cell types and one type of glia in an ordered and overlapping sequence (Fig. 1B and 1C). A series of transcription factors and signaling molecules endow RPCs with competence to generate specific retinal cell types [7]. Intriguingly, many of these regulatory factors sub-serve the same function in other tissues, such as the cerebral cortex, highlighting conserved determination of cell fate across diverse Central Nervous System (CNS) tissues [8]. Transcriptome profiles of developing human retina have provided new insights into temporal and regional cell fate specification by suggesting distinct trajectories of neuronal birth in the fovea versus the peripheral retina [9]. Mutations in retinal developmental genes are key causes of profound pediatric vision loss [10], resulting both in Leber congenital amaurosis (LCA) and juvenile forms of retinitis pigmentosa [11]. Identification of disease-causing genes and elucidation of respective pathogenic mechanisms offer opportunities for developing novel treatment modalities.

## 2 Congenital eye diseases

Congenital eye defects account for up to 60% of blindness among infants and an estimated 1.4 million visually impaired children under the age of 16 worldwide [10,12]. Among over 450 reported clinical manifestations of congenital eye disorders in the Online Mendelian Inheritance in Man database (OMIM; https://omim.org), the etiology of many remains elusive. In this review, we briefly discuss the genetic basis of three common forms of pediatric eye disease – coloboma, congenital glaucoma and LCA and describe current treatment strategies, or those in progress, to alleviate the phenotypes and/or restore vision.

### 2.1 Ocular coloboma

Coloboma is a congenital anomaly which is estimated to account for 11% of pediatric blindness and characterized by an inferior or ventrally located gap in one or more tissues, extending between the cornea and the optic nerve [5,6]. Cases may be unilateral or bilateral, usually with a genetic etiology, and comprise a clinical spectrum that includes congenitally reduced ocular size (microphthalmia), and in severe cases, absence of one or both eyes (anophthalmia). Patients with unilateral anophthalmia and contralateral colobomatous microphthalmia demonstrate that these disorders represent a phenotypic continuum [13]. Although the mechanisms by which coloboma-causing mutations induce unilateral disease remain undefined, their identification is expected to signify a key step in determining therapeutic targets.

**Fig. 2:**
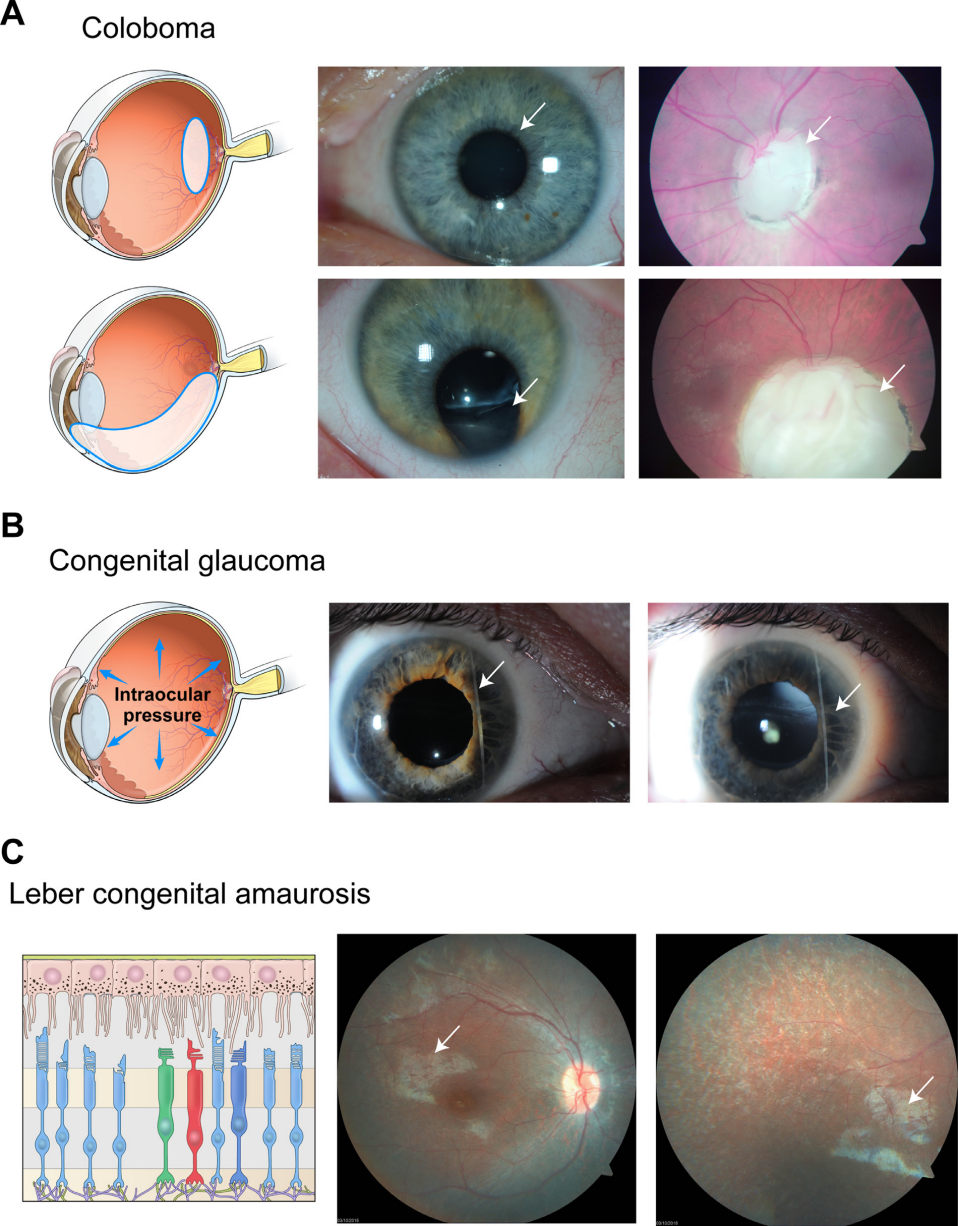
Etiology and phenotypes of pediatric eye diseases. (A) Coloboma. Optic fissure closure defects affecting the formation of the optic nerve (upper) and iris (lower) as shown by graphic illustration (left), clinical images (middle) and fundus images (right). The blue circles and arrows highlight the approximate location of the optic fissure closure defects in the eye. In the upper panel, there is colobomatous enlargement of the optic nerve without iris involvement. In contrast, both the iris and the retina are impacted in the lower panel. (B) Congenital glaucoma. Increased intraocular pressure causes corneal enlargement with splits in Descemet's membrane. Also present are a detached posterior embryotoxon (white arrow) and irido-corneal adhesions (middle and right). (C) Leber congenital amaurosis (LCA) is characterized by dysfunction or loss light-sensitive photoreceptors. In this patient, the death of photoreceptors is caused by a loss-of-function mutation of *RPE65* leading to visual cycle defect in the retinal pigment epithelium. White arrows in the fundus images (middle and right) indicate the affected regions.

Coloboma is readily explicable by perturbed morphogenesis – failure of choroid fissure fusion. The severity broadly correlates with involvement of essential retinal structures, such as the macula. Consequently, iris colobomata that primarily intensify light entry are associated with a relatively mild vision impairment (20/30 to 20/60 acuity), while those affecting the retina, and particularly the macula and optic nerve, result in profound reductions in vision (potentially 20/200 to ‘counting fingers’ levels) (Fig. 2A). The last two decades have seen substantial advances in deciphering the genetic bases of coloboma, which is estimated to have a heritability of at least 80% in developed countries. Interestingly, extensive genetic heterogeneity exists, with mutations in almost 40 genes molecularly explaining only a minority of cases (Table 1A). Consequently, elucidation of molecules and pathways involved in optic fissure closure continues to uncover disease-causing coloboma genes.

**Table 1:** Genes implicated in pathogenesis of pediatric eye diseases A Coloboma*

OMIM number	Gene symbol	Protein	Phenotype	Mode of inheritance	Syndromic (S) or isolated (I)
102560	*ACTG1*	Actin gamma 1	Deafness, autosomal dominant 20/26	AD	S
107480	*SALL1*	Spalt like transcription factor 1	Townes-Brocks branchiootorenal-like syndrome; Townes-Brocks syndrome 1	AD	S
107580	*TFAP2A*	Transcription factor AP-2 alpha	Branchiooculofacial syndrome	AD	S
107773	*NR2F2*	Nuclear receptor subfamily 2 group F member 2	46, XX sex reversal 5; Congenital heart defects, multiple types, 4	AD	S
109400	*PTCH2*	Patched 2	Basal cell nevus syndrome	AD	S
109400	*PTCH1*	Patched 1	Basal cell nevus syndrome	AD	S
109400	*SUFU*	SUFU negative regulator of hedgehog signaling	Basal cell nevus syndrome	AD	S
112262	*BMP4*	Bone morphogenetic protein 4	Microphthalmia, syndromic 6; Orofacial cleft 11	AD	S
113620	*TFAP2A*	Transcription factor AP-2 alpha	Branchiooculofacial syndrome	AD	S
120330	*PAX2*	Paired box 2	Papillorenal syndrome	AD	S
120433	*YAP1*	Yes1 associated transcriptional regulator	Coloboma, ocular, with or without hearing impairment, cleft lip/palate, and/or mental retardation	AD	S
132890	*NR2F1*	Nuclear receptor subfamily 2 group F member 1	Bosch-Boonstra-Schaaf optic atrophy syndrome	AD	S
142993	*VSX2*	Visual system homeobox 2	Microphthalmia with coloboma 3; Microphthalmia, isolated 2	AR	I
145410	*GBBB2/ SPECC1L*	Sperm antigen with calponin homology and coiled-coil domains 1 like	Opitz GBBB syndrome, type II	AD	S
147950	*FGFR1*	Fibroblast growth factor receptor 1	Hypogonadotropic hypogonadism 2 with or without anosmia	AD	S
154400	*SF3B4*	Splicing factor 3b subunit 4	Acrofacial dysostosis 1, Nager type	AD	S
154500	*TCOF1*	Treacle ribosome biogenesis factor 1	Treacher Collins syndrome 1	AD	S
156845	*MITF*	Melanocyte inducing transcription factor	COMMAD syndrome; Tietz albinism-deafness syndrome; Waardenburg syndrome, type 2A; Waardenburg syndrome/ocular albinism, digenic	AD or AR	S
157170	*SIX3*	SIX homeobox 3	Holoprosencephaly 2; Schizencephaly	AD	S or I
163200	*NRAS*	NRAS proto-oncogene, GTPase	Schimmelpenning-Feuerstein-Mims syndrome, somatic mosaic	AD	S
163200	*HRAS*	HRAS proto-oncogene, GTPase	Schimmelpenning-Feuerstein-Mims syndrome, somatic mosaic	AD	S
163200	*KRAS*	KRAS proto-oncogene, GTPase	Schimmelpenning-Feuerstein-Mims syndrome, somatic mosaic	AD	S
164950	*FGF3*	Fibroblast growth factor 3	Deafness, congenital with inner ear agenesis, microtia, and microdontia	AR	S
167409	*PAX2*	Paired box 2	Glomerulosclerosis, focal segmental, 7	AD	S
177075	*MAF*	MAF bZIP transcription factor	Cataract 21, multiple types; Ayme-Gripp syndrome	AD	S or I
184429	*SOX2*	SRY-box transcription factor 2	Optic nerve hypoplasia and abnormalities of the central nervous system; Microphthalmia, syndromic 3	AD	S
191170	*TP53*	Tumor protein p53	Adrenocortical carcinoma, pediatric; Basal cell carcinoma 7; Choroid plexus papilloma; Colorectal cancer; Glioma susceptibility 1; Osteosarcoma; Bone marrow failure syndrome 5; Breast cancer, somatic; Hepatocellular carcinoma, somatic; Li-Fraumeni syndrome; Nasopharyngeal carcinoma, somatic; Pancreatic cancer, somatic	AD, SMu	S
200990	*KIF7*	Kinesin family member 7	Joubert syndrome 12; Acrocallosal syndrome; Al-Gazali-Bakalinova syndrome; Hydrolethalus syndrome 2	AR	S
206700	*ITPR1*	Inositol 1,4,5-trisphosphate receptor type 1	Gillespie syndrome	AD or AR	S
206900	*SOX2*	SRY-box transcription factor 2	Optic nerve hypoplasia and abnormalities of the central nervous system; Microphthalmia, syndromic 3	AD	S
213300	*INPP5E*	Inositol polyphosphate-5-phosphatase E	Joubert syndrome 1	AR	S
216360	*TMEM67*	Transmembrane protein 67	COACH syndrome 1	AR	S
216820	*SALL2*	Spalt like transcription factor 2	Coloboma, ocular	AR	I
218340	*C12orf57*		Temtamy syndrome	AR	S
221900	*ATOH7*	Atonal bHLH transcription factor 7	Persistent hyperplastic primary vitreous	AR	I
235730	*ZEB2*	Zinc finger E-box binding homeobox 2	Mowat-Wilson syndrome	AD	S
243310	*ACTB*	Actin beta	CHARGE syndrome; Hypogonadotropic hypogonadism 5 with or without anosmia	AD	S
243605	*CENPF*	Centromere protein F	Stromme syndrome	AR	S
244450	*UBE3B*	Ubiquitin protein ligase E3B	Single median maxillary central incisor	AD	S
248190	*CLDN19*	Claudin 19	Hypomagnesemia 5, renal, with ocular involvement	AR	S
248390	*POLR1C*	RNA polymerase I and III subunit C	Treacher Collins syndrome 3	AR	S
248450	*FREM1*	FRAS1 related extracellular matrix 1	Manitoba oculotrichoanal syndrome	AR	S
263750	*DHODH*	Dihydroorotate dehydrogenase (quinone)	Miller syndrome	AR	S
270420	*SPINT2*	Serine peptidase inhibitor, Kunitz type 2	Diarrhea 3, secretory sodium, congenital and chorioretinal coloboma	AR	S
274270	*DPYD*	Dihydropyrimidine dehydrogenase	Dihydropyrimidine dehydrogenase deficiency; 5-fluorouracil toxicity	AR	S
300166	*BCOR*	BCL6 corepressor	Microphthalmia, syndromic 2	XLD	1
300244	*FLNA*	Filamin A	Terminal osseous dysplasia	XLD	S
300307	*GZF1*	GDNF inducible zinc finger protein 1	Cleft palate with ankyloglossia	AR	S
300463	*PQBP1*	Polyglutamine binding protein 1	Renpenning syndrome	XLR	S
300472	*IGBP1*	Immunoglobulin binding protein 1	Corpus callosum, agenesis of, with mental retardation, ocular coloboma and micrognathia	XLR	S
300915	*HMGB3*	High mobility group box 3	Microphthalmia, syndromic 13	XL	S
309500	*PQBP1*	Polyglutamine binding protein 1	Renpenning syndrome	XLR	S
309800	*NAA10*	N-alpha-acetyltransferase 10, NatA catalytic subunit	Microphthalmia, syndromic 1	XL	I
600037	*OTX2*	Orthodenticle homeobox 2	Microphthalmia, syndromic 5; Retinal dystrophy, early-onset, with or without pituitary dysfunction; Pituitary hormone deficiency, combined, 6	AD	I
600251	*SPECC1L*	Sperm antigen with calponin homology and coiled-coil domains 1 like	Facial clefting, oblique, 1	AD	S
600463	*ALDH1A3*	Aldehyde dehydrogenase 1 family member A3	Microphthalmia, isolated 8	AR	I
600725	*SHH*	Sonic hedgehog signaling molecule	Holoprosencephaly 3; Microphthalmia with coloboma 5; Schizencephaly; Single median maxillary central incisor	AD	S or I
601147	*GDF6*	Growth differentiation factor 6	Klippel-Feil syndrome 1;	AD or AR	S
601147	*GDF6*	Growth differentiation factor 6	Microphthalmia with coloboma 6, digenic; Leber congenital amaurosis 17; Microphthalmia, isolated 4; Multiple synostoses syndrome 4	AD or AR	I
601186	*STRA6*	Signaling receptor and transporter of retinol STRA6	Microphthalmia, isolated, with coloboma 8, or syndromic 9	AR	S or I
601707	*SMO*	Smoothened, frizzled class receptor	Curry-Jones syndrome, somatic mosaic	Unclear	S
601723	*FZD5*	Frizzled class receptor 5	Microphthalmic coloboma	AD	I
601723	*FZD5*	Frizzled class receptor 5	Microphthalmic coloboma	AD	I
602000	*POLR1B*	RNA polymerase I subunit B	Treacher-Collins syndrome 4	AD	S
602457	*FADD*	Fas associated via death domain	Infections, recurrent, with encephalopathy, hepatic dysfunction, and cardiovascular malformations	AR	S
603714	*SIX3*	SIX homeobox 3	Holoprosencephaly 2; Schizencephaly	AD	S or I
604219	*CRYAA*	Crystallin alpha A	Cataract 9, multiple types	AD or AR	I
604294	*VAX1*	Ventral anterior homeobox 1	Microphthalmia, syndromic 11	AR	I
605124	*SPINT2*	Serine peptidase inhibitor, Kunitz type 2	Diarrhea 3, secretory sodium, congenital and chorioretinal coloboma	AR	S
605452	*ABCB6*	ATP binding cassette subfamily B member 6	Microphthalmia, isolated, with coloboma 7; dyschromatosis universalis hereditaria 3; pseudohyperkalemia, familial, 2, due to red cell leak	AD	S or I
605947	*PIGL*	Phosphatidylinositol glycan anchor biosynthesis class L	CHIME syndrome	AR	S
606522	*GDF3*	Growth differentiation factor 3	Microphthalmia with coloboma 6, or isolated 7; Klippel-Feil syndrome 3	AD	S or I
607086	*AAT1*	Aortic aneurysm, familial thoracic 1	Aortic aneurysm, familial thoracic 1	Unknown	S
607108	*PAX6*	Paired box gene 6	Coloboma of optic nerve; Coloboma, ocular; Morning glory disc anomaly; Aniridia; Anterior segment dysgenesis 5, multiple subtypes; Cataract with late-onset corneal dystrophy; Foveal hypoplasia 1; Keratitis; Optic nerve hypoplasia	AD	I
607906	*ALG2*	ALG2 alpha-1,3/1,6-mannosyltransferase	Congenital disorder of glycosylation, type Ii	AR	S
608166	*SEMA3E*	Semaphorin 3E	CHARGE syndrome	AD	S
608296	*FIBP*	FGF1 intracellular binding protein	Thauvin-Robinet-Faivre syndrome	AR	S
608488	*SMOC1*	SPARC related modular calcium binding 1	Microphthalmia with limb anomalies	AR	S
608553	*NMNAT1*	Nicotinamide nucleotide adenylyltransferase 1	Leber congenital amaurosis 9	AR	I
608572	*TXNL4A*	Thioredoxin like 4A	Burn-McKeown syndrome	AR	S
608892	*CHD7*	Chromodomain helicase DNA binding protein 7	CHARGE syndrome; Hypogonadotropic hypogonadism 5 with or without anosmia	AD	S
608944	*FREM1*	FRAS1 related extracellular matrix 1	Bifid nose with or without anorectal and renal anomalies; Manitoba oculotrichoanal syndrome; Trigonocephaly 2	AD or AR	S
609637	*ZIC2*	Zic family member 2	Holoprosencephaly 5	AD	I
610256	*FOXE3*	Forkhead box E3	Anterior segment dysgenesis 2, multiple subtypes	AR	I
610688	*TMEM67*	Transmembrane protein 67	Joubert syndrome 6	AR	S
610745	*STRA6*	Signaling receptor and transporter of retinol STRA6	Microphthalmia, isolated, with coloboma 8, or syndromic 9	AR	S or I
610937	*RPGRIP1L*	RPGRIP1 like	COACH syndrome 3; Joubert syndrome 7; Meckel syndrome 5	Unknown	S
611254	*KIF7*	Kinesin family member 7	Joubert syndrome 12; Acrocallosal syndrome; Al-Gazali-Bakalinova syndrome; Hydrolethalus syndrome 2	AR	S
611499	*GUSB*	Glucuronidase beta	Mucopolysaccharidosis VII	AR	S
612109	*HMX1*	H6 family homeobox 1	Oculoauricular syndrome	AR	S
612379	*SRD5A3*	Steroid 5 alpha-reductase 3	Congenital disorder of glycosylation, type Iq	AR	S
612713	*SRD5A3*	Steroid 5 alpha-reductase 3	Kahrizi syndrome	AR	S
612779	*DPYD*	Dihydropyrimidine dehydrogenase	Dihydropyrimidine dehydrogenase deficiency; 5-fluorouracil toxicity	AR	S
613001	*FGFR1*	Fibroblast growth factor receptor 1	Encephalocraniocutaneous lipomatosis, somatic mosaic	Unknown	S
613456	*ALX1*	ALX homeobox 1	Frontonasal dysplasia 3	AR	S
613477	*PDE6D*	Phosphodiesterase 6D	Developmental and epileptic encephalopathy 5	AD	S
613477	*PDE6D*	Phosphodiesterase 6D	Developmental and epileptic encephalopathy 5	AD	S
613517	*MCOP6/ PRSS56*	Serine protease 56	Microphthalmia, isolated 6	AR	I
613674	*SOX17*	SRY-box transcription factor 17	Vesicoureteral reflux 3	AD	S
613717	*POLR1D*	RNA polymerase I and III subunit D	Treacher Collins syndrome 2	AD or AR	S
613842	*GZF1*	GDNF inducible zinc finger protein 1	Joint laxity, short stature, and myopia	AR	S
614583	*ACTG1*	Actin gamma 1	Baraitser-Winter syndrome 2	AD	S
615009	*PACS1*	Phosphofurin acidic cluster sorting protein 1	Schuurs-Hoeijmakers syndrome	AD	S
615113	*ALDH1A3*	Aldehyde dehydrogenase 1 family member A3	Microphthalmia, isolated 8	AR	I
615140	*C12orf57*		Temtamy syndrome	AR	S
615145	*TENM3*	Teneurin transmembrane protein 3	Microphthalmia, isolated, with coloboma 9, or syndromic 15	AR	S or I
615147	*RBP4*	Retinol binding protein 4	Retinal dystrophy, iris coloboma, and comedogenic acne syndrome	AR	S or I
615665	*PDE6D*	Phosphodiesterase 6D	Joubert syndrome 22	AR	S
615665	*PDE6D*	Phosphodiesterase 6D	Joubert syndrome 22	AR	S
615877	*MAB21L2*	Mab-21 like 2	Microphthalmia/coloboma and skeletal dysplasia syndrome	AD, AR	S
615877	*MAB21L2*	Mab-21 like 2	Microphthalmia/coloboma and skeletal dysplasia syndrome	AD or AR	S or I
616428	*RBP4*	Retinol binding protein 4	Microphthalmia, isolated, with coloboma 10	AD	I
616490	*KIAA0586*	KIAA0586	Joubert syndrome 23	AR	S
616722	*MIR204*	microRNA 204	Retinal dystrophy and iris coloboma with or without cataract	AD	I
616789	*MED13L*	Mediator complex subunit 13L	Mental retardation and distinctive facial features with or without cardiac defects	AD	S
617107	*FIBP*	FGF1 intracellular binding protein	Thauvin-Robinet-Faivre syndrome	AR	S
617662	*GZF1*	GDNF inducible zinc finger protein 1	Joint laxity, short stature, and myopia	AR	S
618586	*WDR37*	WD repeat domain 37	Neurooculocardiogenitourinary syndrome	AD	S
618652	*WDR37*	WD repeat domain 37	Neurooculocardiogenitourinary syndrome	AD	S
618939	*POLR1B*	RNA polymerase I subunit B	Treacher-Collins syndrome 4	AD	S
619113	*RPGRIP1L*	RPGRIP1 like	COACH syndrome 3; Joubert syndrome 7; Meckel syndrome 5	Unknown	S

*There are 317 entries in OMIM for coloboma. This table only lists those with strong association to coloboma or diseases with coloboma as one of the major clinical characteristics. AD, autosomal dominant; AR, autosomal recessive; DR, digenic recessive; SMu, Somatic mutation; XL, X-linked; XLD, X-linked dominant; XLR, X-linked recessive.

Ocular morphogenesis is guided by combinatorial interactions of transcription factors and gradients of signaling molecules; therefore, such components are frequently associated with coloboma (and of micro/anophthalmia) (Table 1A). For instance, *Pax2* and *Pax6* have essential antagonistic roles in the dorsal-ventral partitioning of the developing optic vesicle, respectively delineating the optic stalk and optic cup. Mutations of *PAX2* induce optic nerve colobomata (and renal anomalies), while *PAX6* mutations can lead to an extensive anomaly spectrum that includes coloboma and microphthalmia [14]. Similarly, perturbation of every phase of eye development, from eye field specification through migration of retinal progenitor cells and axis formation to the migration of the neural crest-derived periocular mesenchyme, may impair choroid fissure closure. Indeed, reflecting the fundamental nature of these processes, causative mutations have now been identified in members of most developmental pathways such as those corresponding to Hedgehog, RA, Bone morphogenetic protein (BMP), TGF-β, Fibroblast growth factor (FGF), Wnt and Hippo signaling [6,15]. The main exception appears to be Notch signaling, where ligand mutation induces coloboma in murine but leads to discrete anterior segment phenotypes in patients [16]. Genes involved in cell proliferation/migration/death signaling pathways are also involved in epithelial remodeling at the fissure, yet apoptosis has not been detected by a recent transcriptome analysis of optic fissure closure signature genes using human samples, thereby suggesting distinctions among species [15,17]. It is also important to highlight that the process of tissue fusion required for choroid fissure closure is not unique to the eye and occurs at multiple sites including the neural tube, palate and lip. Likely reflecting evolutionary parsimony, genetic pathways implicated in coloboma, neural tube defects and cleft palate are largely conserved and therefore treatment strategies developed in one tissue may have applicability to others.

### 2.2 Anterior segment dysgenesis

The anterior segment of the eye comprises the tissues (from cornea to lens) that lie in front of the vitreous. Their key roles include refracting and focusing incident light onto the retina and circulating aqueous humor, which is essential for maintaining clarity of the avascular cornea and lens. Maldevelopment of the anterior segment frequently results in early-onset glaucoma. One of the subtypes, congenital glaucoma, is characterized by chronic intraocular pressure (IOP) elevation. Affected infants exhibit ocular enlargement which manifests as increased corneal diameter and frequently splits in Descemet's membrane (Haab striae) [18] as well as increased lacrimation (Fig. 2B). Additional features include angle anomalies, IOP elevation to 30–50 mm of mercury, and optic disc cupping that may be partially reversible with prompt normalization of IOP [19]. Iris alterations are observed in some molecular subtypes, along with a range of systemic anomalies in syndromic cases. Clinical management of the patients is usually surgical and is reviewed in [20].

Although exhibiting some common etiologies with coloboma, congenital glaucoma can be caused by alteration of transcription factors or signaling pathways crucial for the development of the anterior segment. From a genetics standpoint, the congenital glaucoma phenotype is intriguing for its molecular diversity, which encompasses alterations in RA, TGF-β and angiopoietin signaling as well as transcriptional networks involving *PAX6, FOXC1* and *PITX2* [21], [22], [23], [24], [25] (Table 1B). These developmental pathways control the formation of anterior segment of the eye, and their perturbation impairs aqueous humor drainage and induces IOP elevation. A majority of cases can be attributed to mutations in *CYP1B1* [26,27], which encodes a cytochrome P450 oxygenase for stepwise conversion of retinol (vitamin A) to retinal and then to RA [28]. Biallelic *CYP1B1* mutations result in near complete loss of function of this RA-synthesizing enzyme and thus disrupt RA morphogen gradients needed for the genesis of anterior segment, perturbing the peri-ocular mesenchyme from which the ciliary body, trabecular meshwork, iris, corneal stroma and endothelium are derived. RA also directly regulates *FOXC1* and *PITX2* [29], two transcription factors with fundamental roles in anterior segment development [21,23,24], likely explaining why ocular anomalies induced by *FOXC1* and *PITX2* mutations can phenocopy congenital glaucoma.

As a recessively inherited disorder, the incidence of congenital glaucoma is strongly influenced by geographic prevalence of consanguinity. Consanguineous cases are frequently bilateral, and more often associated with greater disease severity. In these patients, higher IOPs and the associated decrease in corneal clarity may preclude angle surgery (called goniotomy), necessitating more complex surgical approaches. Non-consanguineous cases are more often unilateral and amenable to goniotomy, suggesting that modifier loci may contribute to phenotypic severity. Notably, intermediate levels of RA resulting from a heterozygous *CYP1B1* mutation induce milder disease, with such mutations explaining 5% of the related juvenile open-angle glaucoma phenotypes diagnosed between 5 and 40 years of age [30]. Thus, studies of early-onset and comparatively extreme pediatric phenotypes exemplify the power to decipher genetic mechanisms in common later-onset diseases.

A similar paradigm is illustrated by angiopoietin-Tie (ANG-TIE) signaling pathway, an essential regulator of blood and lymphatic development, in the genesis of congenital glaucoma. Mouse mutants with conditional deletion of the Angiopoietin 1 and 2 (Angpt1/2) ligands that bind to the TIE receptors develop ocular enlargement and optic neuropathy, in addition to complete absence of Schlemm's canal, a modified lymphatic that drains aqueous humor from the eye [31]. Heterozygous *TIE2* mutations cause ~5% of congenital glaucoma [32], with a few disrupting TIE2 receptor clustering that is required for pathway activation [33]. *TIE2* haploinsufficiency can recapitulate variable expressivity of *CYP1B1*-mediated congenital glaucoma in terms of age of onset and unilateral disease prevalence [32]. Subsequent investigations have identified *ANGPT1* mutations in congenital glaucoma and, equally importantly, suggest the requirement of ANG-TIE signaling for maintaining the Schlemm's canal [34,35]. Intriguingly, aging-associated decline in functional integrity of Schlemm's canal can be rescued by an agonistic Tie2 antibody, at least in mice [34]. In light of Schlemm's canal's central role, additional ANG-TIE components are predicted to be involved in glaucoma pathogenesis, offering novel targets for intervention.

### 2.3 Leber congenital amaurosis

In vertebrates, photons are captured by light-sensitive photoreceptors in the neural retina, integrated and processed by interneurons (the horizontal, bipolar and amacrine cells) and transmitted to the brain through the optic nerve composed of the retinal ganglion cell axons and glia (Fig. 1B). Vision loss stems from obstruction of the light path to the neural retina and inability of the retina to detect and/or transmit light-triggered signals to the brain. In retinal degenerative diseases, the irreversible blindness is largely attributable to dysfunction or death of photoreceptor cells [11] (Fig. 2C). Amongst such disorders, LCA is a group of severe and early-onset retinal diseases responsible for childhood blindness [36]. LCA patients exhibit profound visual impairment at infancy or in childhood, with almost undetectable full field electroretinogram, nystagmus (eye movements), poor pupillary light responses, and oculodigital reflex [37],[38]. LCA accounts for approximately 5% of all retinal dystrophies, affecting 2–3 per 100,000 live births with an estimated prevalence of 180,000 individuals worldwide [37].

**Table 1b:** Congenital glaucoma#

OMIM number	Gene symbol	Protein	Phenotype	Mode of inheritance	Syndromic (S) or isolated (I)
105650	*RPS19*	Ribosomal protein S19	Diamond-Blackfan anemia 1	AD	S
137600	*PITX2*	Paired like homeodomain 2	Anterior segment dysgenesis 4	AD	I
175780	*COL4A1*	Collagen type IV alpha 1 chain	Brain small vessel disease with or without ocular anomalies including congenital glaucoma	AD	S
180849	*CREBBP*	CREB binding protein	Rubinstein-Taybi syndrome 1	AD	S
236670	*POMT1*	Protein O-mannosyltransferase 1	Muscular dystrophy-dystroglycanopathy (congenital with brain and eye anomalies), type A, 1; Muscular dystrophy-dystroglycanopathy (congenital with mental retardation), type B, 1; Muscular dystrophy-dystroglycanopathy (limb-girdle), type C, 1	AR	S
249420	*SH3PXD2B*	SH3 and PX domains 2B	Frank-ter Haar syndrome with or without glaucoma	AR	S
251750	*LTBP2*	Latent transforming growth factor beta binding protein 2	Weill-Marchesani syndrome 3; Glaucoma 3, primary congenital, D; Microspherophakia and/or megalocornea, with ectopia lentis and with or without secondary glaucoma	AR	S or I
253280	*POMGNT1*	Protein O-linked mannose N-acetylglucosaminyltransferase 1 (beta 1,2-)	Muscular dystrophy-dystroglycanopathy (congenital with brain and eye anomalies), type A, 3	AR	S
600221	*TEK*	TEK receptor tyrosine kinase	Glaucoma 3, primary congenital, E; Venous malformations, multiple cutaneous and mucosal	AD	S or I
601090	*FOXC1*	Forkhead box C1	Anterior segment dysgenesis 3, multiple subtypes; Axenfeld-Rieger syndrome, type 3	AD	S or I
601631	*FOXC1*	Forkhead box C1	Anterior segment dysgenesis 3, multiple subtypes; Axenfeld-Rieger syndrome, type 3	AD	S or I
601652	*MYOC*	Myocilin	Glaucoma 1A, primary open angle	AD	I
601771	*CYP1B1*	Cytochrome P450 family 1 subfamily B member 1	Anterior segment dysgenesis 6, multiple subtypes; Glaucoma 3A, primary open angle, congenital, juvenile, or adult onset	AR	I
602091	*LTBP2*	Latent transforming growth factor beta binding protein 2	Weill-Marchesani syndrome 3; Glaucoma 3, primary congenital, D; Microspherophakia and/or megalocornea, with ectopia lentis and with or without secondary glaucoma	AR	S or I
603474	*RPS19*	Ribosomal protein S19	Diamond-Blackfan anemia 1	AD	S
604563	*SBF2*	SET binding factor 2	Charcot-Marie-Tooth disease, type 4B2	AR	S
607423	*POMT1*	Protein O-mannosyltransferase 1	Muscular dystrophy-dystroglycanopathy (congenital with brain and eye anomalies), type A, 1; Muscular dystrophy-dystroglycanopathy (congenital with mental retardation), type B, 1; Muscular dystrophy-dystroglycanopathy (limb-girdle), type C, 1	AR	S
610192	*GLIS3*	GLIS family zinc finger 3	Diabetes mellitus, neonatal, with congenital hypothyroidism. Additional features include congenital glaucoma	AR	S
610199	*GLIS3*	GLIS family zinc finger 3	Diabetes mellitus, neonatal, with congenital hypothyroidism. Additional features include congenital glaucoma	AR	S
613150	*POMT2*	Protein O-mannosyltransferase 2	Muscular dystrophy-dystroglycanopathy (congenital with brain and eye anomalies), type A, 2	AR	S
613293	*SH3PXD2B*	SH3 and PX domains 2B	Frank-ter Haar syndrome with or without glaucoma	AR	S
617315	*CYP1B1*	Cytochrome P450 family 1 subfamily B member 1	Anterior segment dysgenesis 6, multiple subtypes; Glaucoma 3A, primary open angle, congenital, juvenile, or adult onset	AR	I

#There are 55 entries in Online Mendelian Inheritance in Man (OMIM, https://www.ncbi.nlm.nih.gov/omim) for congenital glaucoma. This table only lists those with strong association to congenital glaucoma or diseases with congenital glaucoma as one of the major clinical features. AD, autosomal dominant; AR, autosomal recessive; XLR, X-linked recessive.

**Table 1c:** Leber Congenital Amaurosis (LCA)^

OMIM number	Gene symbol	Protein	Phenotype	Mode of inheritance	Syndromic (S) or isolated (I)
204000	*GUCY2D*	Guanylate cyclase 2D, retinal	Leber congenital amaurosis 1	AD or AR	I
601777			Cone-rod dystrophy 6		
204100	*RPE65*	Retinoid isomerohydrolase RPE65	Leber congenital amaurosis 2	AR	I
613794			Retinitis pigmentosa 20		
618697			Retinitis pigmentosa 87 with choroidal involvement	AD	
604232	*SPATA7*	Spermatogenesis associated 7	Leber congenital amaurosis 3; Retinitis pigmentosa, juvenile	AR	I
604393	*AIPL1*	Aryl hydrocarbon receptor interacting protein like 1	Cone-rod dystrophy; Retinitis pigmentosa, juvenile; Leber congenital amaurosis 4	AD or AR	I
604537	*LCA5*	Lebercilin LCA5	Leber congenital amaurosis 5	AR	I
613826	*RPGRIP1*	RPGR interacting protein 1	Leber congenital amaurosis 6	AR	I
613829	*CRX*	Cone-rod homeobox	Leber congenital amaurosis 7	AD	I
613835	*CRB1*	Crumbs cell polarity complex component 1	Leber congenital amaurosis 8	AR	I
600105			Retinitis pigmentosa-12		
608553	*NMNAT1*	Nicotinamide nucleotide adenylyltransferase 1	Leber congenital amaurosis 9	AR	I
611755	*CEP290*	Centrosomal protein 290	Leber congenital amaurosis 10	AR	I or S
610188			Joubert syndrome 5		
610189			Senior-Loken syndrome 6		
613837	*IMPDH1*	Inosine monophosphate dehydrogenase 1	Leber congenital amaurosis 11	AR	I
180105			Retinitis pigmentosa 10	AD	
610612	*RD3*	RD3 regulator of GUCY2D	Leber congenital amaurosis 12	AR	I
612712	*RDH12*	Retinol dehydrogenase 12	Leber congenital amaurosis 13	AD or AR	I
613341	*LRAT*	Lecithin retinol acyltransferase	Retinitis pigmentosa, juvenile; Leber congenital amaurosis 14; Retinal dystrophy, early-onset severe	AR	I
613843	*TULP1*	TUB like protein 1	Leber congenital amaurosis 15	AR	I
600132					
614186	*KCNJ13*	Potassium inwardly rectifying channel subfamily J member 13	Leber congenital amaurosis 16	AR	I
615360	*GDF6*	Growth differentiation factor 6	Leber congenital amaurosis 17	AR	I
608133	*PRPH2*	Peripherin 2	Leber congenital amaurosis 18; Retinitis pigmentosa 7 and digenic form	AD or AR	I or S
169150			Macular dystrophy, patterned, 1		
618513	*USP45*	Ubiquitin specific peptidase 45	Leber congenital amaurosis 19	AR	I
617879	*TUBB4B*	Tubulin beta 4B class IVb	Leber congenital amaurosis with early-onset deafness	AD	S
609237	*IQCB1*	IQ motif-containing protein B1	Leber congenital amaurosis	AR	I or S
609254			Senior-Loken syndrome 5	AR	

^There are 101 entries in OMIM (https://www.ncbi.nlm.nih.gov/omim) for LCA. This table only lists the genes with strong association to LCA or diseases with LCA as one of the major clinical attributes. AD, autosomal dominant; AR, autosomal recessive; DR, digenic recessive; XL, X-linked.

Molecular diagnosis of LCA can be challenging because of phenotypic variability and genetic heterogeneity. Mutations in at least 28 genes have been reported to cause 70-75% of LCA, which is largely inherited as an autosomal recessive trait though dominant mutations have been reported in *IMPDH1, OTX2* and *CRX* genes [39] (Table 1C). LCA genes can be categorized in discrete functional classes, including transcriptional regulation and primary cilia (the photoreceptor outer segment is a modified cilium) biogenesis and transport. Pleiotropic effects of mutations in LCA genes add another level of genetic complexity; e.g., defects in cilia-associated genes (such as *CEP290*) can lead to syndromic diseases that usually include photoreceptor degeneration [40]. Genetic modifiers can also influence clinical features of ciliopathy gene defects; the phenotype of *CEP290* mutations is amenable modifications by its interaction partners [41,42]. Although the *rd16* mouse model phenocopies *CEP290*-LCA with rapid degeneration of photoreceptors [43], a humanized knock-in mouse model carrying the most common *CEP290*-LCA mutation (c.2991 + 1655A>G) does not show obvious phenotypes [44], suggesting unique mechanisms of disease pathogenesis in humans. Notably, retinal organoids differentiated from induced pluripotent stem cells (iPSCs) of a *CEP290*-LCA patient demonstrate disease-associated cilia findings *in vitro* [45]. Persistence of photoreceptor cell bodies in some LCA patients with advanced disease, despite undetectable visual function, suggest possibilities of vision restoration by targeted therapies [38].

## 3 Therapeutic strategies

As a transparent, compartmentalized and immune-privileged organ, it is self-evident why the eye offers exceptional opportunities for evaluating different treatment paradigms. However, designing treatment for congenital eye disease is still challenging. This reflects clinical presentation months after the period of perturbed *in utero* development, the involvement of multiple ocular tissues and cell types, as well as tremendous genetic heterogeneity. Each of the three diseases discussed here poses unique complexities regarding the timing and methods of delivery to the therapeutic targets. Retinal dystrophies are currently the most tractable for correction, benefitting from many decades of fundamental research in retinal genetics and cell biology, and the retina comprising of a single tissue. Despite its fiendishly complex neuronal composition and synaptic organization, the retina has been a focus for therapeutic trials because of its unique accessibility within the CNS. In contrast, congenital glaucoma involves anterior and posterior segment tissues, requiring therapeutic modification of multiple cell types including dysfunctional and dying lateral geniculate nucleus-projecting retinal ganglion cells. The challenges are further augmented in colobomata, which at a minimum manifest many months after arising during early development. Prevention of colobomata requires the identification of disease-causing genetic profiles and their precise correction before the initiation of the optic fissure closure at gestation week 16; such corrections are not feasible currently since tissue regeneration would be required at an unprecedented scale, with existing stem cells coaxed into repopulating the affected area. Cell transplantation represents another theoretical approach; however, it is unclear whether signaling cues required for donor cell engraftment, division and differentiation persist to recapitulate such lengthy periods of normal development. As a result, the following section largely focuses on therapeutic approaches developed in retinal disorders (Table 2), which provide a broader exemplar for the CNS, and that in time may be extended to other pediatric eye disorders. Considerable progress has been made, in part, because of a longer window of intervention opportunity for retinal diseases.

**Table 2:** Pros and cons of major therapeutic approaches

	Successful examples	Strengths	Limitations
Gene therapy	• FDA approved the first gene therapy drug Luxturna for RPE65-LCA • CRISPR/Cas9-mediated genome editing precisely corrected disease-causing mutations in preclinical models of CEP290-LCA • Antisense oligonucleotide-based therapy partially restored CEP290-LCA patient vision in one clinical trial	• High specificity to mutations and relevant tissues • Clear evidence of clinical efficacy • Low risk of immune response in the eyes	• Packing limit of adeno-associated viruses (AAV) • Complexity and cost of manufacturing and production • Unclear effect of long-term expression of genome editors or augmented genes • Off-target effect of genome editors • Accessibility to correct early-onset congenital diseases
Large and small molecule drugs	• Readthrough drug PCT124 in Phase II clinical trial for treatment of *PAX6*-coloboma • Therapeutic antibody against vascular endothelial growth factor (VEGF) in Phase IV clinical trial for treatment of age-related macular degeneration	*Small molecule* • Ease in administration and dosage control • High scale of synthesis • Low cost • Be able to cross blood barriers or placenta • Be able to target multiple tissues simultaneously *Large molecule (antibody)* • High specificity • High stability	• Low tissue specificity • High demand on physiology-relevant models to evaluate pharmacological effects and pharmacokinetics
Cell replacement therapy	• Transplantation of stem cell-derived retinal pigment epithelium in preclinical and clinical trials for age-related macular degeneration	• Large treatment window	• Consistency and quality of cell-based therapies • Safety and ethical issues of cell source • Exchange of cytoplasmic materials between the host and graft cells

### 3.1 Gene therapy

Gene therapy harnesses different vectors/vehicles for delivering desired gene products into affected tissues and/or cell types. A widely used approach for gene delivery in eye tissues is the use of viral vectors simply by injection at the preferred site and with low risk of immune response [46]. The low rate of integration into the host genome makes adeno-associated viral (AAV) vectors a promising platform for gene therapy [47]. One successful example of this approach is the first FDA-approved drug for treatment of LCA caused by *RPE65* loss-of-function mutations [38,48]; however, we should mention that the long-term data from clinical trials have been less encouraging [49]. A second potentially exciting approach is CRISPR/Cas9-mediated genome editing [50], which can potentially correct disease-causing mutations in multiple scenarios (from retinal explants, humanized mice, non-human primates to patient iPSC-derived retinal organoids) [51]. However, the technique is currently constrained by limited editing efficiency [52] and off-target mutations that include induced chromosomal anomalies [53]. Another promising methodology is use of antisense oligonucleotides (AON) [54], which induce quite persistent suppression of pathological RNA transcripts by exon skipping and other mechanisms. Several of these are in therapeutic use for pediatric neurological disorders such as Duchenne Muscular Dystrophy and Spinal Muscular Atrophy, while AON-based therapy for *CEP290*-LCA has yielded encouraging results [55], with vision improvement without serious adverse effect reported in one clinical trial [56].

A key limitation of gene therapy for congenital eye diseases is the temporal window for effective treatment. AAV vectors cannot reach the target cells of fetus, and a vast majority of early-onset disorders already exhibit severe developmental defects or cell loss at birth [5]. The small packaging limit of AAV (<5kb) also restricts its application for diseases caused by larger genes. In such cases, alternative approaches include gene augmentation by delivering parts of the gene [57,58], use of lentiviral vectors with larger packaging capacity [59], or splitting the transgene into two separate AAV vectors [60]; however, the efficiency and/or safety of these approaches in humans require further investigations. In any case, it would be time-consuming and currently prohibitively expensive to tailor gene therapy for each causative mutation, particularly since a therapy effective for one mutation may not be readily extrapolated to phenotypes caused by another [61]. Thus, innovative mutation-independent strategies are needed to maintain cell survival or restore visual function. One encouraging example is provided by CRISPR-mediated knockdown of a key transcriptional regulator *Nrl*, which has generated longer survival of (dysfunctional) rod photoreceptors while preserving the cones that are essential for the macula and thus fine vision [62]. Viral-mediated expression of rod-derived cone viability factor (RdCVF) also shows promising effect in the maintenance of cone and rod photoreceptors in multiple mouse models of retinal degeneration [63]. Optogenetic therapies deliver light-activated ion channels to surviving retinal cell types (bipolar cell and retinal ganglion cells), restoring a degree of photosensitivity in animal models [64,65]; and clinical trials are in progress. In a related manner, engraftment of optogenetically engineered photoreceptors has achieved partial recovery of visual function in the murine retina [66]. Such results are naturally very encouraging; however, it remains to be determined whether this promise translates into long-lasting restoration of retinal function in humans.

### 3.2 Large and small molecule drugs

Small molecule drugs offer an effective and widely used approach, reflecting ease in administration and dosage control, stability, scale of synthesis and low cost [67]. Importantly, the ability of many small molecule drugs to cross the blood-brain (or even placental) barrier may facilitate treatment of early-onset ocular/neurological diseases. Pharmaceutical modulation of signaling pathways contributing to cell death is a more specific way to preserve their survival and function [68]. Photoswitchable ion channel blockers can also offer potential for restoring vision [69]. Small molecule drugs have been applied for reading through a stop codon, correcting the structure of mutated proteins or circumventing functions of abnormal proteins [5,70]. Notably, the readthrough drug PCT124 was reported to be effective in a *Pax6* mutation iris coloboma model, leading to initiation of a phase II clinical trial [5]. Such approaches hold great potential for treating developmental defects caused by misregulation of signaling pathways, such as the ANG-TIE signaling pathway for congenital glaucoma. Antioxidants (e.g., vitamin A, vitamin B3, docosahexaenoic acid, lutein), anti-apoptotic factors (e.g., tauroursodeoxycholic acid, rasagiline, norgestrel, and myriocin) and neurotrophic factors (e.g., ciliary neurotrophic factor (CNTF), Brain-derived neurotrophic factor (BDNF)) have been evaluated in the treatment of retinal degenerative diseases [40]. Therapeutic antibodies have been extensively used to neutralize bioactive factors, as illustrated by intravitreally administered monoclonals to vascular endothelial growth factor (VEGF) that are effective in treatments of neovascular age-related macular degeneration [71].

A major challenge for developing relevant drug targets is identification of appropriate molecules with excellent pharmacological benefit and pharmacokinetics and low off-target effects [67], especially in case of small molecules that can penetrate various tissues. However, ninety percent of drug candidates fail to progress from Phase I trials to clinical use [72], partly because a majority of the drugs are identified using adherent cell culture or small animal models, which, although offering valuable mechanistic insights, do not fully recapitulate human pathobiology. Recent advances in three-dimensional human retinal organoids that structurally and functionally, at least in part, mimic *in vivo* tissues can provide a promising platform for complementing the existing strategies for identifying drug candidates [73]. A recent breakthrough of deep-learning program for determining three-dimensional shapes of proteins without crystallography should accelerate the process of drug design and discovery [74].

### 3.3 Cell replacement therapy

When affected cells are lost or grossly abnormal at infancy, regenerative medicine may offer a plausible approach for restoring at least partial vision. A few attempts have been made to stimulate regeneration of lost cells from other cell types [75,76], whereas others have generated desired cell types from pluripotent stem cells and transplanted the products into the eye [77]. In LCA and early-onset retinal degeneration, the need to replace photoreceptors for restoring vision requires donor cell survival, maturation (including development of the outer segment) and functional integration to form synapses with host retinal interneurons. Transplantation of photoreceptors was previously demonstrated to improve visual function in animal models, yet recent studies indicate transfer of cytoplasmic material between the donor and host cells, potentially offering unanticipated opportunities for therapeutic delivery [73,78]. In contrast, transplantation of stem cell-derived retinal pigment epithelium that can be produced at high efficiency and purity offers hope in preclinical and clinical trials for age-related macular degeneration [79,80]. In congenital glaucoma, the loss of retinal ganglion cells (RGCs) requires the elongation of axons, integration into the optic nerve and projection to the lateral geniculate nucleus. Despite efficient generation of functional RGCs from pluripotent stem cells, transplantation of these cells has yet to yield desirable results, with extensive investigations continuing in preclinical models [81].

A major concern in using iPSC-derived products is related to genomic stability [82]. Although no adverse effects are reported at this early stage, possible deleterious outcomes, such as tumor formation or degeneration of transplanted cells, may take a longer time frame to unravel. Embryonic stem cells have raised ethical concerns and may trigger immune response, whereas the use of iPSCs may not be feasible for congenital diseases. CRISPR-based approaches have also been utilized for correcting mutations in patient iPSC-derived retinal organoids [83]. Given the immense interest in stem cells, rapid advancements are expected in generation of photoreceptors from stem cells [84] and in gene-editing. Further investigations are necessary to overcome numerous challenges for correct functional integration of transplanted cells into the host retina with minimal undesirable consequences [85].

## Outstanding questions

Several questions remain. Can we accelerate gene discovery for pediatric eye diseases? Is high variability of congenital eye phenotypes caused by gene/pathway interactions? How to efficiently and safely design prevention or treatment paradigms for congenital diseases *in utero*? Is it possible to selectively manipulate signaling pathways to expedite and improve the specificity of treatments? What would criteria/standards be for devising such therapies for distinct early onset diseases affecting the children?

## Search strategy and selection criteria

The references were identified using Google Scholar and PubMed search engines with the search terms “coloboma” or “congenital glaucoma” or “Leber congenital amaurosis” or “congenital eye diseases” or “coloboma AND genetics” or “congenital glaucoma AND genetics” or “Leber congenital amaurosis AND genetics” or “gene therapy AND congenital eye diseases” or “small molecule AND congenital eye diseases” or “cell replacement therapy AND congenital eye diseases” or “human eye development” or “treatment AND congenital diseases”. Disease-associated genes were identified by the Online Mendelian Inheritance in Man database (OMIM; https://www.ncbi.nlm.nih.gov/omim) using keywords “coloboma” or “congenital glaucoma” or “Leber congenital amaurosis” and further selected using Google Scholar and Genecards engines with the search terms “[gene name] AND [disease]”. All references were considered with preference for the most recently published works.

## Conclusions

Congenital ocular diseases exhibit extensive genotypic and phenotypic heterogeneity. Despite identification of many disease-causing genes, genetic defects remain to be discovered in a large number of congenital eye diseases. We believe a gene-independent approach will be desirable for developing therapies of such a divergent cohort. A network-based approach by modulating pathways associated with congenital eye diseases and a combination of gene and small-molecule based therapies would likely have promising impact on treating early-onset eye diseases. Nonsense suppression therapy, neurotrophic and antiapoptotic as well as other small molecule drugs might help in maintaining the survival of defective cells and achieve at least a partially desirable treatment outcome.

## Declaration of Competing Interest

All authors declare that they have no competing interests.
